# Identification and Characterization of Segregation Distortion Loci on Cotton Chromosome 18

**DOI:** 10.3389/fpls.2016.02037

**Published:** 2017-01-12

**Authors:** Baosheng Dai, Huanle Guo, Cong Huang, Muhammad M. Ahmed, Zhongxu Lin

**Affiliations:** National Key Laboratory of Crop Genetic Improvement, College of Plant Science and Technology, Huazhong Agricultural UniversityWuhan, China

**Keywords:** cotton, segregation distortion, gametic competition, zygotic selection, maternal effect

## Abstract

Segregation distortion is commonly detected via genetic mapping and this phenomenon has been reported in many species. However, the genetic causes of the segregation distortion regions in a majority of species are still unclear. To genetically dissect the SD on chromosome 18 in cotton, eight reciprocal backcross populations and two F_2_ populations were developed. Eleven segregation distortion loci (SDL) were detected in these ten populations. Comparative analyses among populations revealed that SDL18.1 and SDL18.9 were consistent with male gametic competition; whereas SDL18.4 and SDL18.11 reflected female gametic selection. Similarly, other SDL could reflect zygotic selection. The surprising finding was that SDL18.8 was detected in all populations, and the direction was skewed towards heterozygotes. Consequently, zygotic selection or heterosis could represent the underlying genetic mechanism for SDL18.8. Among developed introgression lines, SDL18.8 was introgressed as a heterozygote, further substantiating that a heterozygote state was preferred under competition. Six out of 11 SDL on chromosome 18 were dependent on the cytoplasmic environment. These results indicated that different SDL showed varying responses to the cytoplasmic environment. Overall, the results provided a novel strategy to analyze the molecular mechanisms, which could be further exploited in cotton interspecific breeding programs.

## Introduction

Segregation distortion (SD) is defined as a deviation of the observed allelic frequencies at a locus from the expected Mendelian ratio in a segregating population. This phenomenon is commonly detected via genetic mapping and has been documented in various species, including mouse (Eversley et al., 2010; Casellas et al., 2012), *Drosophila* (Phadnis and Orr, 2009; Larracuente and Presgraves, 2012; McDermott and Noor, 2012), *Tigriopus* (Pritchard et al., 2011), rice (Koide et al., 2012; Reflinur et al., 2014; Xu et al., 2014), maize (Tang et al., 2013), and cotton (Yu et al., 2011; Hulse-Kemp et al., 2015). SD, a powerful evolutionary force, has been suggested as a selection mechanism among different gametophyte and/or sporophyte genotypes (Sandler and Novitski, 1957). Moreover, SD could be involved in the alleviation of population divergence leading to speciation (McDermott and Noor, 2010).

Several factors could affect gametophyte and zygote formation and ultimately lead to SD. Several genetic mechanisms of SD have been insightfully studied in plants and animals (Larracuente and Presgraves, 2012; Yang et al., 2012). For example, zygotic selection rather than gametic selection might play an important role in SD in diploid alfalfa (Li et al., 2011). However, both male gametic and zygotic selection contributed to the severe SD of a locus during maternal haploid induction in maize (Xu et al., 2013). Furthermore, meiotic drive could increase the frequency of distorted alleles, which eventually become fixed in the population. A sex ratio distortion has previously been reported in mosquito via meiotic drive (Shin et al., 2012). In addition, conspecific pollen precedence has been recognized as a potential major source for SD in closely related species of *Mimulus* with divergent mating systems (Fishman et al., 2008).

Molecular markers with SD are typically distributed in clusters and are primarily skewed in the same direction; these regions are generally defined as segregation distortion regions (SDRs) (Lu et al., 2002; Eversley et al., 2010; Li et al., 2010; Leppala et al., 2013). For example, in maize, 14 SDRs were detected among 9 different chromosomes, and 4 SDRs were located in the vicinity of gametophyte genes, suggesting that these SDRs might be partially induced by gametophyte genes (Yan et al., 2003). Lu et al. (2002) reported that 18 chromosomal regions on 10 maize chromosomes were associated with SD, and three known gametophytic factors were potential genetic stimulants of these SDRs. In barley, a total of 14 SDRs have been identified, and the association of the identified SDRs and haploid production genes were compared (Li et al., 2010).

The most prospective explanation for the SDRs could be that specific loci in the genome are conduced to viability differentiation (Luo and Xu, 2003; Zhu and Zhang, 2007). The selection of an allele at the locus would result in nearby markers that deviate from the expected ratio, consistent with the theory of genetic hitchhiking. Thus, analysis of the mapped molecular markers in the vicinity along the genome would be helpful to analyze segregation distortion loci (SDL). Based on the genotypic frequency of the markers, Luo et al. (2005) developed a quantitative genetics model for mapping SDL, assuming a continuous liability that controls the viability of individuals. Subsequently, an SDL mapping module based on the EM (expectation-maximization) algorithm was integrated in PROC QTL software, making this method friendly to use (Xu and Hu, 2009).

In a previous study, our laboratory constructed a cotton interspecific genetic linkage map that included 2316 loci on 26 chromosomes using a BC_1_ population of 141 individuals (Yu et al., 2011). A total of 21 SDRs were detected, with 5 SDRs on chromosome 18, and the molecular markers on chromosome 18 were severely distorted. However, thus far, little is known about the genetic mechanism of SD on chromosome 18 in cotton. In the present study, eight reciprocal backcross populations and two F_2_ populations were developed to reveal SDRs, primarily focusing on the exploitation of the genetic mechanism of SD in severely distorted chromosome 18. We investigated the marker segregation in the ten populations, and subsequently we identified SDL using Proc QTL. Moreover, we substantiated the putative genetic mechanism underlying these SDL.

## Materials and Methods

### Plant Materials

**FIGURE 1:**
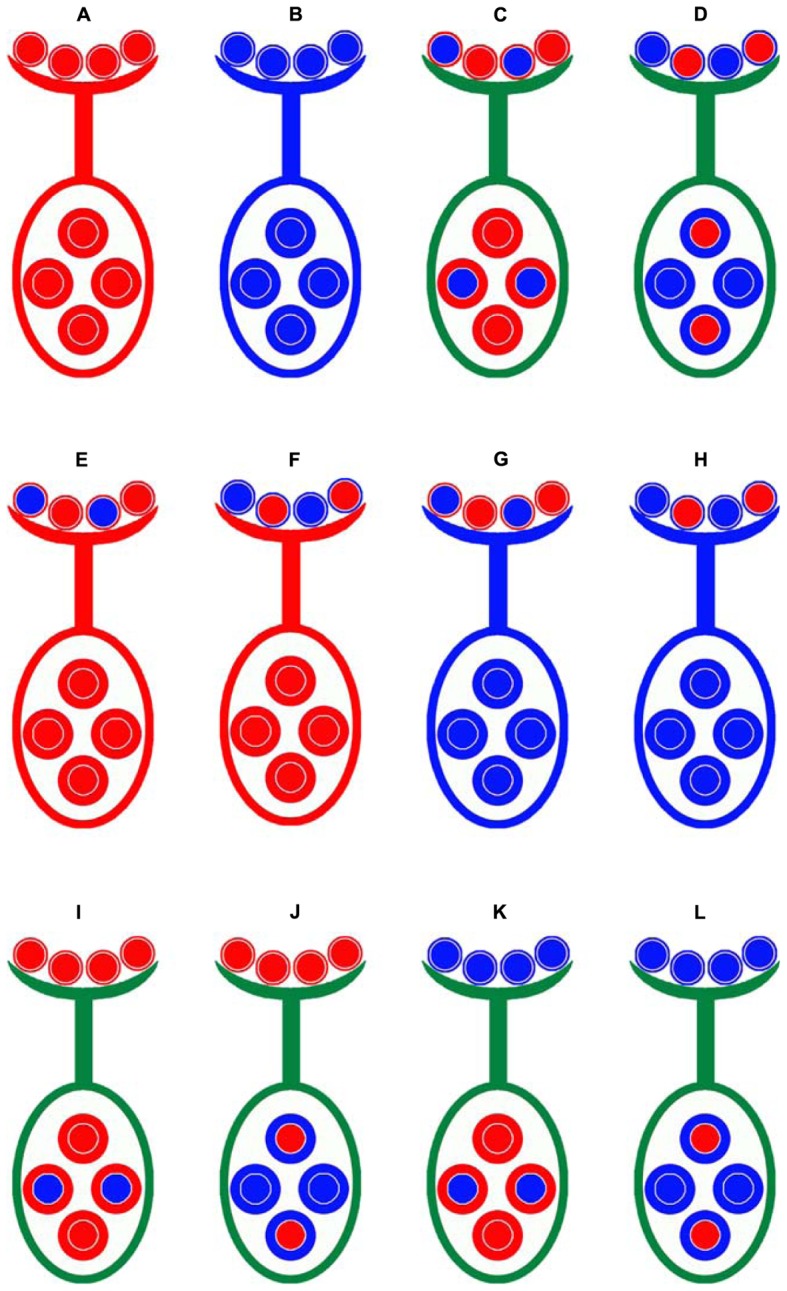
**Crossing design for distinguishing gametic and zygotic selection and the maternal effects for SD.** Emian22 **(A)** was crossed with 3-79 **(B)** to produce two types of F_1_ plants, these F_1_ hybrid plants were self-pollinated to generate F_2_ populations, (E3)F_2_ **(C)** and (3E)F_2_ **(D)**. Four male-segregating backcross populations, E(E3) **(E)**, E(3E) **(F)**, 3(E3) **(G)**, and 3(3E) **(H)**, and four female-segregating backcross populations, (3E)E **(I)**, (E3)E **(J)**, (3E)3 **(K)**, and (E3)3 **(L)**, were developed. Smaller circles indicate pollen on stigma and larger circles indicate ovules. The colors of patterns outside of the two types of circles are indicated as the cytoplasmic backgrounds, red is indicated for Emian22, and blue is indicated for 3-79.

The *Gossypium hirsutum* cv. Emian22 and *G. barbadense* acc. 3–79 were used as the parents to develop eight reciprocal BC_1_F_1_ populations and two F_2_ populations (**Figure 1**). Emian22 is an elite cultivar cultivated in Hubei province, China; and 3-79 is considered as a genetic and cytogenetic standard line for *G. barbadense* (Yu et al., 2011).

Emian22 was crossed with 3-79 to produce reciprocal F_1_ plants, namely, Emian22/3-79 and 3-79/Emian22, and the reciprocal F_1_ hybrid plants were self-pollinated to generate the F_2_ mapping populations (Emian22/3-79) F_2_ and (3-79/Emian22) F_2_, designated (E3)F_2_ and (3E)F_2_, respectively. Subsequently, two sets of reciprocal BC_1_F_1_ populations were developed in the present study. The F_1_ plant derived from the cross Emian22/3-79 was backcrossed with the Emian22 or 3-79, and the pedigrees of these populations are Emian22 // (Emian22/3-79), (Emian22/3-79) // Emian22, 3-79 // (Emian22/3-79) and (Emian22/3-79) // 3-79, designated E(E3), (E3)E, 3(E3), and (E3)3, respectively. Additionally, the other four reciprocal BC_1_F_1_ populations were produced by backcrossing the F_1_ derived from 3-79/Emian22 and Emian22 or 3-79, and the pedigrees of these populations are Emian22 // (3-79/Emian22), (3-79/Emian22) // Emian22, 3-79 // (3-79/Emian22) and (3-79/Emian22) // 3-79, designated E(3E), (3E)E, 3(3E), and (3E)3, respectively.

The following numbers of progeny from the BC_1_F_1_ populations and F_2_ were used for mapping and SD analysis: 142 for (E3)E, 142 for (E3)3, 142 for (3E)E, 142 for (3E)3, 190 for E(E3), 190 for E(3E), 142 for 3(E3), 138 for 3(3E), 142 for (3E)F_2_, and 142 for (E3)F_2_. All plant materials were planted during the cotton-growing season in 2012 at the experimental farm of Huazhong Agricultural University, Wuhan, China.

### Molecular Marker Genotyping

Total genomic DNA from the parents and individuals of the eight BC_1_F_1_ and two F_2_ populations were extracted from young leaves according to Paterson et al. (1993). To compare the population difference, co-dominant markers were selected to genotype the ten populations. A total of fifty polymorphic molecular markers covering 136.9 cM along chromosome 18 were genotyped in the ten populations. The primer sequences of the molecular markers were obtained from CottonGen^1^ (Yu et al., 2014). Polymerase chain reaction (PCR) analysis, electrophoresis and silver staining were performed according to Lin et al. (2005).

### SDL Detection

For each locus, deviations from the Mendelian ratios (1:1 ratio for BC_1_F_1_ population, and 1:2:1 for F_2_ population) were estimated and examined for significance using chi-square analysis. To account for multiple testing, the Benjamini-Hochberg False Discovery Rate (FDR) correction method was applied to the segregation data of each population to avoid type-I errors deriving from the large number of tests. The method was performed calling the *p.adjust* function incorporated in the R program STATS. To avoid false positives, the adjusted p-values were used to determine significance. The loci showing non-Mendelian segregation (*P* < 0.05) were considered to exhibit SD. The SD of an individual marker could reflect linkage to an SDL. The identification of candidate regions containing SDL is an effective method to resolve the genetic architecture of SD. The EM (expectation–maximization) method used for mapping SDL in a segregating population provided an efficient approach to estimate the positions and effects of putative SDL in the genetic map (Xu and Hu, 2009). SDL were detected by using the PROC QTL according to the method of Xu and Hu (2009). The detailed procedure is available in the PROC QTL manual^2^. In the output result table, the loci were designated as significant SDL with a LOD value of 3.0 (Tang et al., 2013).

### Identification and Annotation of Genes in the SDL

To investigate the genes in these SDL, the sequences containing SSRs were acquired from CottonGen (Yu et al., 2014). Using the BLASTX (Altschul et al., 1990), these sequences were mapped to the cotton genome (TM-1) (Zhang et al., 2015), and the physical positions of these SDL were identified. The genes in the adjacent region were obtained for every SDL. Further, gene ontology (GO) enrichment analysis was investigated using Fisher’s exact test in Blast2Go with a cut-off *E*-value of 0.001 (Conesa et al., 2005). Blast2Go was used to compare the frequency of the GO terms in the reference genes with the cotton genome and the test genes.

## Results

### Patterns of Marker Segregation Across the Chromosome 18

**FIGURE 2:**
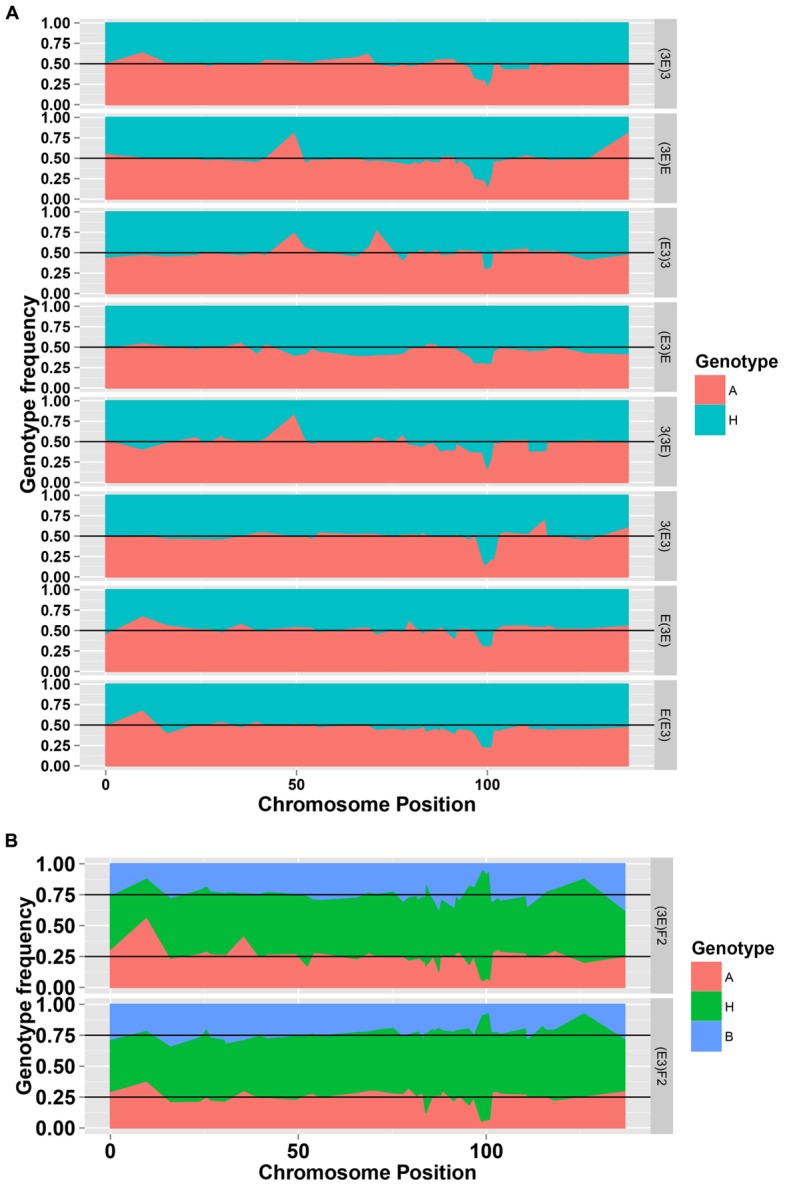
**The genotype frequencies of the SSR markers on chromosome 18 in the eight BC_1_F_1_ populations** **(A)** and two F_2_ populations **(B)**.

**FIGURE 3:**
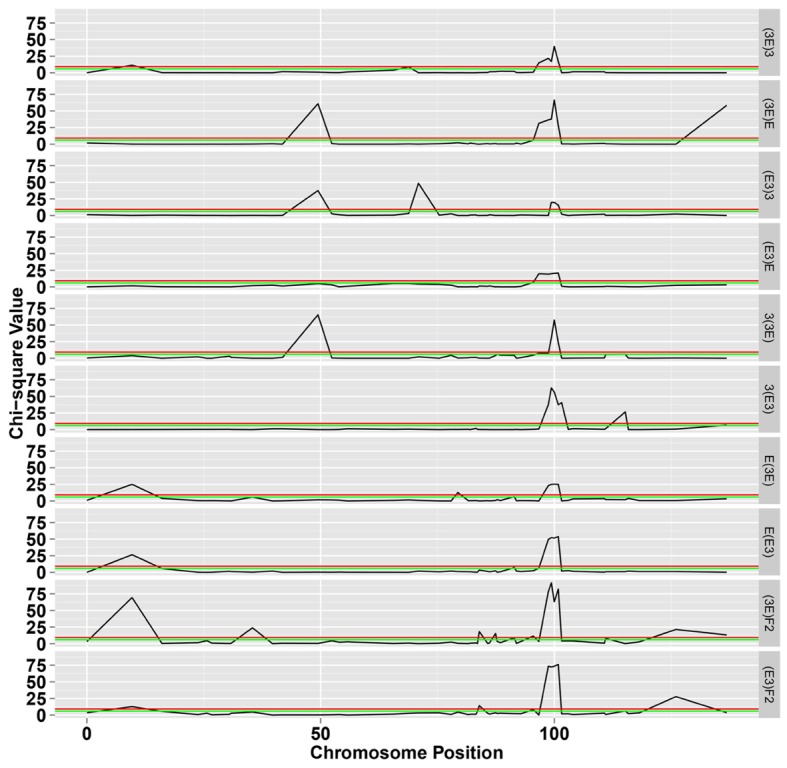
**The chi-square test for segregation of Mendelian inheritance on chromosome 18.** The green and black lines represent the significance level at 0.05 and 0.01, respectively.

To facilitate a comparative analysis between populations, a total of 50 co-dominant SSR markers were used to genotype these ten populations; dominant markers were not used. **Figures 2** and **3** show the frequencies and chi-square values of the two genotypes of SSR markers on chromosome 18 in all populations. According to the Mendel’s segregation laws, the genotype ratio at a locus should be 1:1 in BC_1_F_1_ populations and 1:2:1 in F_2_ populations; however, there could be several severely distorted regions along chromosome 18.

The markers of distorted segregation in the eight reciprocal BC_1_F_1_ populations and two F_2_ populations are presented in **Table 1**. The results indicated that a total of 17 markers showed distorted segregation. Among these markers, three markers, HAU1908, MUSS140 and NAU3398 were synchronously distorted in all populations. The region spanning 99.368–100.854 cM showed continuous SD. In addition, the adjacent markers, NAU3232 and HAU2992, were distorted in several populations. NAU2980a was synchronously distorted in the three BC_1_F_1_ populations and the (3E)F_2_ population. Similarly, JESPR178 was synchronously distorted in the three BC_1_F_1_ populations, and HAU2631a was synchronously distorted in the two F_2_ populations. Similarly, TMB2762 was distorted in the two BC_1_F_1_ populations. The other markers were distorted in only one population.

**Table 1:** Markers of distorted segregation along chromosome 18.

Markers	Position	%EE	33%	%EE	33%	%EE	33%	%EE	33%	%EE	%EE
		(E3)E	(E3)3	(3E)E	(3E)3	E(E3)	E(3E)	3(E3)	3(3E)	(3E)F_2_	(E3)F_2_
NAU2980a	9.647				0.65^∗^	0.69^∗∗^	0.68^∗∗^			0.57^∗∗^(EE)	
NAU4861	35.413									0.42^∗∗^(EE)	
JESPR178	49.432		0.76^∗∗^	0.83^∗∗^					0.85^∗∗^		
HAU1381	68.842				0.64^∗^						
NAU5364	70.926		0.80^∗∗^								
BNL1040	79.354						0.63^∗∗^				
JESPR153	83.945									0.18^∗^(H)	
BNL2652	91.411					0.40^∗^					
NAU3816	95.48										0.18^∗^(H)
NAU3232	96.693	0.31^∗∗^		0.26^∗∗^	0.33^∗∗^						
HAU2992	98.711	0.31^∗∗^		0.24^∗∗^	0.30^∗∗^	0.24^∗∗^	0.32^∗∗^	0.23^∗∗^		0.07^∗∗^(H)	0.06^∗∗^(H)
HAU1908	99.368	0.31^∗∗^	0.31^∗∗^	0.24^∗∗^	0.32^∗∗^	0.23^∗∗^	0.32^∗∗^	0.15^∗∗^	0.26^∗∗^	0.06^∗∗^(H)	0.06^∗∗^(H)
MUSS140	99.98	0.30^∗∗^	0.31^∗∗^	0.15^∗∗^	0.23^∗∗^	0.24^∗∗^	0.31^∗∗^	0.17^∗∗^	0.17^∗∗^	0.08^∗∗^(H)	0.06^∗∗^(H)
NAU3398	100.854	0.30^∗∗^	0.33^∗∗^	0.27^∗∗^	0.32^∗∗^	0.23 ^∗∗^	0.32^∗∗^	0.23^∗∗^	0.29^∗∗^	0.06^∗∗^(H)	0.07^∗∗^(H)
BNL3558	101.558							0.22^∗∗^			
BNL193	115.174							0.72^∗∗^			
HAU2631a	126.015									0.20^∗∗^(H)	0.25^∗∗^(H)
TMB2762	136.867			0.82^∗∗^				0.62^∗^			


*Genotypic ratios were tested against the expected Mendelian expectation to determine the significant of SD. ^∗^*P* < 0.05, ^∗∗^*P* < 0.01 (Bonferroni’s corrected using the *p.adjust* function incorporated in the R program STATS). EE and 33 are abbreviations for homozygote ‘Emian22’ and ‘3-79’, respectively. The letters in the parenthesis denote the skew direction, and H is the abbreviation for heterozygote ‘3E’.*

The patterns of marker segregation were different in each population, indicating that the genetic mechanism of SD had distinct population specificity as a result of complex genetic systems. Among these 17 distorted markers, ten markers were skewed towards heterozygotes and seven markers were skewed towards homozygotes, indicating that heterozygotes were transmitted at a higher frequency than homozygotes.

### SDL Detection Along the Chromosome 18

**FIGURE 4:**
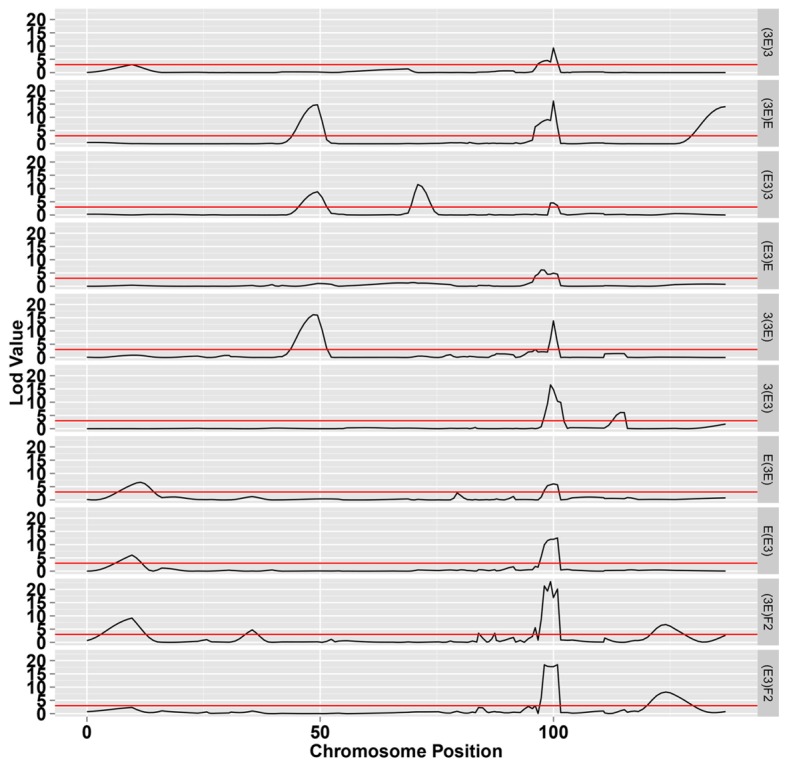
**The LOD score for segregation distortion loci (SDL) on chromosome 18.** The black line represents the significance level at LOD value = 3.0.

The LOD profiles for the detected SDL are presented in **Figure 4**, and peaks with LOD scores more than 3.0 indicated the presence of SDL. Eleven SDL were detected in all populations, and details of these SDL are presented in **Table 2**. SDL18.1, located at 9.65 cM on chromosome 18, was detected in the E(E3), E(3E), and (3E)F_2_ populations; SDL18.2, located at 35.41 cM, was detected in the (3E)F_2_ populations; SDL18.3, located at 49.432 cM, was detected in the (E3)3, (3E)E, and 3(3E) populations; SDL18.4, located at 70.962 cM, was detected in the (E3)3 population; SDL18.5 and SDL18.6, located at 83.95 cM, 87.41 cM, respectively, were detected in the (3E)F_2_ populations; SDL18.7, located at 95.48 cM, was detected in both F_2_ populations; SDL18.8, located at 99.98 cM, was detected in all populations; SDL18.9, located at 115.174 cM, was detected in the 3(E3) population; SDL18.10, located at 126.02 cM, was detected in both F_2_ populations; and SDL18.11, located at 136.867 cM, was detected in the (3E)E population. The results presented in the present study vividly demonstrated that the regions contained certain genetic factors, which could be responsible for the SD on chromosome 18.

**Table 2:** Segregation distortion loci (SDL) detected along chromosome 18 in the 10 populations.

SDL	Adjacent marker	Position (cM)	Population	LOD	33/3E/EE (%)	χ^2^ value
SDL18.1	NAU2980a	9.65	E(E3)	6.06	NA/0.31/0.69	26.53
			E(3E)	6.67	NA/0.32/0.68	25.06
			(3E)	9.18	0.58/0.31/0.11	69.47
SDL18.2	NAU4861	35.41	(3E)	4.73	0.42/0.35/0.23	23.90
SDL18.3	JESPR178	49.432	(E3)3	8.79	0.24/0.76/NA	37.53
			(3E)E	14.71	NA/0.17/0.83	60.91
			3(3E)	11.14	0.85/0.15/NA	65.4
SDL18.4	NAU5364	70.926	(E3)3	11.52	0.80/0.20/NA	48.51
SDL18.5	JESPR153	83.95	(3E)	3.47	0.18/0.68/0.14	18.16
SDL18.6	CM63	87.41	(3E)	3.46	0.13/0.5/0.37	15.34
SDL18.7	NAU3816	95.48	(3E)	5.56	0.19/0.65/0.16	11.57
			(E3)	3	0.19/0.62/0.19	8.19
SDL18.8	MUSS140	99.98	(E3)E	4.92	NA/0.70/0.30	20.36
			(E3)3	4.57	0.31/0.69/NA	19.78
			(3E)E	16.14	NA/0.85/0.15	66.26
			(3E)3	9.3	NA/0.23/0.77	39.61
			E(E3)	12.53	NA/076/0.24	51.58
			E(3E)	6.07	NA/0.69/0.31	25.32
			3(E3)	16.54	0.17/0.83/NA	56.49
			3(3E)	9.65	0.17/0.83/NA	57.4
			(3E)	20.14	0.06/0.88/0.06	82.15
			(E3)	18.47	0.06/0.86/0.08	73.40
SDL18.9	BNL193	115.174	3(E3)	6.07	0.72/0.28/NA	26.58
SDL18.10	HAU2631a	126.02	(3E)	6.73	0.20/0.68/0.12	21.42
			(E3)	8.12	0.25/0.68/0.06	27.76
SDL18.11	TMB2762	136.867	(3E)E	14.03	NA/0.18/0.82	58.32


*EE, 33, and 3E are abbreviations for homozygote ‘Emian22’ and ‘3-79’, heterozygote ‘3E’, respectively.*

Among these SDL, six loci including SDL18.6, SDL18.7, SDL18.8, SDL18.9, SDL18.10, and SDL18.11, have previously been reported (Yu et al., 2011). SDL18.6, SDL18.7, and SDL18.8 were positioned in the previous SDR18.3, which spanned 85.727–102.297 cM; SDL18.9 was positioned in the previous SDR18.4, which spanned 110.264–115.838 cM; and SDL18.10 and SDL18.11 were positioned in the previous SDR18.5, which spanned 126.015–136.867 cM.

### Characterization of SDL Underlying Gametic Selection or Zygotic Selection

SDL18.1 was distorted in the two male-segregating backcross populations, E(E3) and E(3E), and the (3E)F_2_ population. In backcross populations, the direction was skewed towards Emian22 homozygotes, suggesting that in competition, pollens containing the Emian22 allele were preferred compared with pollens containing the 3-79 allele; but it was not distorted in the reciprocal female-segregating backcross populations, (E3)E and (3E)E, implying that this SD reflected male gametic competition. In addition, SDL18.1 was not distorted in the other male-segregating backcross populations, 3(E3) and 3(3E), suggesting that this SD reflected stigma and embryo sac effects. In the (3E)F_2_ population, the skew direction was skewed towards 3-79 homozygotes, implying that zygotic selection might contribute to this SD, i.e., 3-79 homozygotes were preferred in the zygotic embryo stage.

SDL18.2 was detected only in the (3E)F_2_ population, suggesting that this SD resulted from zygotic selection. However, this locus was not distorted in the (E3)F_2_ population, indicating that nucleocytoplasmic interactions could affect this SD; namely, this SD only occurred in embryo sacs with 3-79 cytoplasm. The direction was skewed towards 3-79 homozygotes in the (E3)3 population, suggesting that the 3-79 homozygote was preferred in the zygotic embryo stages under competition.

SDL18.3 was also distorted in the female-segregating backcross populations, (E3)3 and (3E)E, and the male-segregating backcross population, 3(3E), suggesting that this SD might result from zygotic selection. However, SDL18.3 was not distorted in the other five backcross populations, indicating that nucleocytoplasmic interactions could affect this SD. The direction was skewed towards homozygotes in these three populations, suggesting that homozygotes were preferred under competition.

SDL18.4 was detected only in one backcross female-segregating population, (E3)3, and was not distorted in the reciprocal male-segregating backcross population, 3(E3), implying that this SD resulted from female gametic selection. However, this locus was not distorted in the female-segregating population, (3E)3, indicating that nucleocytoplasmic interactions could affect this SD; namely, this SD only occurred in the embryo sacs with Emian22 cytoplasm. The direction was skewed towards homozygotes in the (E3)3 population, suggesting that female gametes containing the 3-79 allele were preferred under prezygotic competition.

SDL18.5 and SDL18.6 were only distorted in (3E)F_2_ population and not in all the backcross populations, implying that this SD was resulted from zygotic selection. Furthermore, this locus was not distorted in the reciprocal (E3)F_2_ population, indicating that the maternal environment could affect this SD; namely, this SD only occurred in the F_1_ plants having 3-79 cytoplasm.

SDL18.7 was distorted in both F_2_ populations, but was not distorted in all the backcross populations, implying that this SD resulted from zygotic selection. Furthermore, the direction was skewed towards heterozygotes, suggesting the preference of this genotype in the zygotic embryo stage under competition.

SDL18.8 was detected in all the backcross and F_2_ populations, implying that this SD resulted from zygotic selection. The direction was skewed towards heterozygotes in these populations, suggesting that heterozygotes were preferred under competition, and consequently, zygotic selection, such as the differentiation of zygote viability or heterosis, may be the genetic mechanism for the observed SD.

SDL18.9 was distorted in only one backcross male-segregating population, 3(E3), but was not distorted in the reciprocal female-segregating backcross population, (E3)3, and F_2_ populations, implying that this SD resulted from male gametic selection. In addition, this locus was not distorted in the male-segregating population, 3(3E), indicating that nucleocytoplasmic interactions could affect this SD; namely, this SD only occured in the pollen mother cells with Emian22 cytoplasm. The direction was skewed towards homozygotes in the 3(E3) population, suggesting that pollens containing the 3-79 allele were preferred under competition compared with pollens containing the Emian22 allele.

SDL18.10 was coincidentally distorted in the two F_2_ populations but was not distorted in all the backcross populations, implying that this SD resulted from zygotic selection. In addition, the direction was skewed towards heterozygote, suggesting that the heterozygote was preferred in the zygotic phase.

SDL18.11 was detected only in one of the backcross female-segregating populations, (3E)E, and was not distorted in the reciprocal male-segregating backcross population, E(3E), and F_2_ populations. These results indicated that female gametic competition, resulting in the preferential fertilization or abortion of gametes or zygotes, was the main factor influencing this SD. In addition, this locus was not distorted in the female-segregating population, (E3)E, indicating that nucleocytoplasmic interactions could affect this SD; namely, this SD only occurred in the embryo sacs with 3-79 cytoplasm. The direction was skewed towards homozygotes in the (3E)E population, suggesting that female gametes containing the Emian22 allele were preferred to female gametes containing the 3-79 allele under competition.

To assess the gametic transmission in the progeny resulting from the interspecific hybridization between the two parents, we examined the genotypes of the 337 introgression lines developed through the continuous crossing of the (E22/3-79)F_1_s with Emian22 as the female and the 515 markers used for assisted selection (Li, 2013). Only SDL18.8 was detected in one introgression line (#M219, BC_7_F_3_), and the genotype remained heterozygote (**Supplementary Figure S1**), although this line had been self-pollinated three times. The results indicated that SDL18.8 was heterozygously transmitted, and the heterozygote indeed has a competitive advantage.

### Cytoplasmic Effects on SD

The cross design in the present study enabled us to determine whether SDL were dependent on the cytoplasmic environment. SDL18.2, SDL18.4, SDL18.5, SDL18.6, SDL18.9, and SDL18.11 were distorted in the (3E)F_2_, (E3)3, (3E)F_2_, (3E)F_2_, 3(E3), and (3E)E populations, respectively. However, these loci were not distorted in their corresponding mutual backcross populations, (E3)F_2_, (3E)3, (E3)F_2_, (E3)F_2_, 3(3E), and (E3)E, respectively, suggesting that these SDL were dependent on the specific cytoplasmic environment, i.e.; the cytoplasmic environment had an important effect on these SDL. SDL18.3, SDL18.7, SDL18.8, and SDL18.10 were simultaneously distorted in the male and female segregating backcross populations, indicating that these SDL were independent on the cytoplasmic environment.

### Characterization of Genes in the SDL

Owing to the recently published tetraploid cotton genomes, we identified the genes in the SDL. For the 11 SDL on chromosome 18, the genomic locations were determined in the cotton genome after mapping the sequences of the adjacent markers. Thereafter, the genes in the regions were acquired from the cotton genome (TM-1) (Zhang et al., 2015). A total of 174 genes were identifed among these 11 SDL, and 112 genes were annotated with their predicted function and GO terms (**Supplementary Table S1**). The functional annotation of the genes showed a diversity of molecular functions (F) and biological processes (P).

Compared with randomly selected cotton genes, the genes in these SDL regions were significantly enriched with GO terms within the categories of carbohydrate metabolic process and gene expression (**Table 3**). Importantly, terms related to glycometabolism, including fructose-bisphosphate aldolase activity (GO:0004332), aldehyde-lyase activity (GO:0016832), glycolysis (GO:0006096), and catabolic process (GO:0009056) were also identified.

**Table 3:** Gene ontology (GO) enrichment analysis of the genes in the eleven SDL (*p*-value < 0.01).

GO-ID	GO Term	Category
GO:0004332	fructose-bisphosphate aldolase activity	F
GO:0016832	aldehyde-lyase activity	F
GO:0006096	glycolysis	P
GO:0010467	gene expression	P
GO:0009056	catabolic process	P


## Discussion

With the development of molecular markers, SD has been widely reported in several plant species, particularly in interspecific crosses for genetic mapping, such as rice (Shanmugavadivel et al., 2013), wheat (Takumi et al., 2013; Adamski et al., 2014), chickpea (Ravikumar et al., 2013), *Arabidopsis* (Leppala et al., 2013), coffee (Gartner et al., 2013), soybean (Baumbach et al., 2012), potato (Manrique-Carpintero et al., 2016), *Brassica rapa* (Kitashiba et al., 2016), and *Populus deltoids* (Zhou et al., 2015). SD has previously been reported in interspecific populations of cotton (Guo et al., 2007; Blenda et al., 2012; Byers et al., 2012; Liang et al., 2012; Yu et al., 2013; Diouf et al., 2014; Zhang et al., 2014; Li et al., 2016), intraspecific populations of *G. barbadense* (Wang et al., 2013), intraspecific populations of *G. hirsutum* (Lin et al., 2009; Zhang et al., 2012, 2016; Ning et al., 2014), and intraspecific populations of *G. arboreum* (Li et al., 2007); however, the genetic mechanism of SD in cotton remains unclear.

Considering evolutionary biology, SD is a selection mechanism that may occur in any stage of the life history, including the gametophyte and zygote. In a backcross population with the F_1_ hybrid serving as the male parent, we can rule out female gametic-specific mechanisms from the male/zygotic mechanisms for SD. However, in a backcross population in which the F_1_ hybrid served as the female parent, we can rule out male gametic-specific mechanisms from the female/zygotic mechanisms for SD (Fishman et al., 2008). In the present study, we developed ten populations to dissect the effects of gametophytic and zygotic selection on chromosome 18 in cotton. Eleven SDL were detected in the ten populations, among which, SDL18.1 and SDL18.9 resulted from male gametic competition, and SDL18.4 and SDL18.11 resulted from female gametic selection. The other SDL likely reflected zygotic selection. These results provided a better understanding of the putative mechanism of SD, which has been reported in many plant species (Anhalt et al., 2008; Fishman et al., 2008; Cai et al., 2011; Castro et al., 2011; Diouf and Mergeai, 2012; Tang et al., 2013).

Several agronomic traits related QTL were mapped on cotton chromosome 18, such as fiber strength (*qFS-C18-1*), uniformity (*qFU-C18-1*), micronaire (*qFMi-C18-1*), maturity (*qFMa-C18-1*), lint weight (*qLW-C18-1*), seed index (*qSI-C18-1*), lint percentage (*qLP-C18-1*), and bud opening (Fu et al., 2013). Moreover, the genic male-sterile genes, *ms5, ms6*, and *ms15*, were mapped on chromosomes 12, 26, and 12 in cotton, respectively (Chen et al., 2009). However, no locus related to gametic competition and zygotic selection has been reported on chromosome 18. According to the results of the present study, a total of eleven SDL were detected on chromosome 18. In maize, the SDRs were examined to locate gametophyte genes (Yan et al., 2003). The results indicated several gametophyte genes on chromosome 18. A total of 112 annotated genes were predicted in these 11 SDL after blasting to tetraploid cotton genome sequences (Zhang et al., 2015). GO enrichment analysis showed that a number of the terms were related to glycometabolism, including the pathway of fructose-bisphosphate aldolase activity (GO:0004332), etc. These results indicated that these complicated glycometabolism pathways may contribute to the SD on cotton chromosome 18.

Cytoplasmic effects might be involved in the viability selection of gametes and zygotes because cytoplasm provides an environment for nuclear gene expression and cellular metabolic reactions. Tang et al. (2013) showed that the maternal cytoplasmic environment might be involved in the viability selection of gametes and zygotes resulting from dramatic changes in the genotypic frequencies of the SDL in the two reciprocal cross populations. In the present study, six out of eleven SDL on chromosome 18 were dependent on the cytoplasmic environment, but other SDL were not. These results indicated that the SDL on different locus had different reactions to the cytoplasmic environment.

To broaden the genetic base of *G. hirsutum* germplasm for genetic improvement, interspecific hybridization and introgression between *G. hirsutum* and *G. barbadense* were extensively employed. However, few successes have been reported, primarily reflecting genetic barriers between the two species, including accumulated gene mutations and gene order rearrangements, particularly SD (Zhang et al., 2014). The success of the breeding programs between the two species is highly dependent on understanding the genetic mechanisms of SD, providing guidance for the selection of a suitable female parent, and marker-assisted selection for SDL will avoid the loss of the desired traits (Li et al., 2010). Hence, further studies are needed to increase the current understanding of the genetic molecular mechanisms related to SDL, which would be useful for breeding programs.

## Author Contributions

BD genotyped the molecular markers, analyzed the genetic data and drafted the manuscript. HG, CH, and MA genotyped molecular markers and generated the figures. ZL designed the study and supervised the experiments and analyses. All authors read and approved the final manuscript.

## Conflict of Interest Statement

The authors declare that the research was conducted in the absence of any commercial or financial relationships that could be construed as a potential conflict of interest.

## Footnotes

1 http://www.cottongen.org

2 http://statgen.ucr.edu/software.html
